# The Expression of Transcription Factors in Fetal Lamb Kidney

**DOI:** 10.3390/jdb9020022

**Published:** 2021-06-19

**Authors:** Yuri Nishiya, Kohei Kawaguchi, Kosuke Kudo, Takuya Kawaguchi, Juma Obayashi, Kunihide Tanaka, Kei Ohyama, Hideki Nagae, Shigeyuki Furuta, Yasuji Seki, Junki Koike, Kevin C. Pringle, Hiroaki Kitagawa

**Affiliations:** 1 Division of Pediatric Surgery, School of Medicine, St. Marianna University, 2-16-1, Sugao, Kawasaki 216-8511, Kanagawa, Japan; yuri.nishiya@marianna-u.ac.jp (Y.N.); k3kawaguchi@marianna-u.ac.jp (K.K.); kosuke.kudo@marianna-u.ac.jp (K.K.); t2kawaguchi@marianna-u.ac.jp (T.K.); j2obayashi@marianna-u.ac.jp (J.O.); k3tanaka@marianna-u.ac.jp (K.T.); k2oyama@marianna-u.ac.jp (K.O.); hi-naga@marianna-u.ac.jp (H.N.); its0408@marianna-u.ac.jp (S.F.); skchldrnshp@gol.com (Y.S.); 2 Department of Pathology, School of Medicine, St. Marianna University, Kawasaki 216-8511, Kanagawa, Japan; j2koike@marianna-u.ac.jp; 3 Department of Obstetrics and Gynecology, School of Medicine & Health Science, University of Otago, North Dunedin, Dunedin 9016, New Zealand; kevin.pringle@otago.ac.nz

**Keywords:** kidney, development, WT1, Pax2, Pax8, HNF1β

## Abstract

(1) Background: Renal development involves frequent expression and loss of transcription factors, resulting in the activation of genes. Wilms’ tumor 1 (WT1), hepatocyte nuclear factor-1-beta (HNF1β), and paired box genes 2 and 8 (Pax2 and Pax8) play an important role in renal development. With this in vivo study, we examined the period and location of expression of these factors in renal development. (2) Methods: Fetal lamb kidneys (50 days from gestation to term) and adult ewe kidneys were evaluated by hematoxylin and eosin staining. Serial sections were subjected to immunohistochemistry for WT1, HNF1β, Pax2, and Pax8. (3) Results: Pax2, Pax8, and HNF1β expression was observed in the ureteric bud and collecting duct epithelial cells. We observed expression of WT1 alone in metanephric mesenchymal cells, glomerular epithelial cells, and interstitial cells in the medullary rays and Pax8 and HNF1β expression in tubular epithelial cells. WT1 was highly expressed in cells more proximal to the medulla in renal vesicles and in C- and S-shaped bodies. Pax2 was expressed in the middle and peripheral regions, and HNF1β in cells in the region in the middle of these. (4) Conclusions: WT1 is involved in nephron development. Pax2, Pax8, and HNF1β are involved in nephron maturation and the formation of peripheral collecting ducts from the Wolffian duct.

## 1. Introduction

The development of the mammalian kidney starts from interactions between the Wolffian duct and the metanephric mesenchyme (MM). In response to signals from the MM, the Wolffian duct forms the ureteric bud, which then invades the MM, branching repeatedly. The ureteric bud’s invasion of the MM leads to the aggregation of the metanephric mesenchyme and induces mesenchymal–epithelial transition (MET), which leads to the formation of renal vesicles and C- and S-shaped bodies. S-shaped bodies lead to the formation of proximal tubules, Henle’s loop, and distal tubules, resulting in a fusion with the ureteric bud. Capillary endothelial cells and mesangial cells invade the cleft of S-shaped bodies. S-shaped-bodies differentiate into Bowman’s capsule, and glomerular epithelial cells form the nephron. As the branching of the UB nears its completion, the terminal ends of the UB branches fuse with the distal ends of the developing nephrons and the UB differentiates into the collecting ducts, which drain into the renal pelvis. This process occurs intermittently during the fetal stage, leading to the formation of a mature kidney [1]. In the above-described renal development, the expression and loss of many transcription factors lead to the activation of various genes. Recent studies on the induction of human embryonic stem cells (hESCs) and human-induced pluripotent stem cells (hiPSCs) in the renal tissue identified transcription factors that play an important role in renal development [2]. It was reported that: (i) SIX2, SALL1, WT1, and paired box gene 2 (Pax2)-positive nephron progenitor cells (NPCs) are induced by hESCs and hiPSCs through the primitive streak and posterior intermediate mesoderm; and (ii) the NPCs result in pretubular aggregates and paired box gene 8 (Pax8), LHX1, and LAM-positive renal vesicles with the expression of hepatocyte nuclear factor-1-beta (HNF1β) and BRN1, subsequently inducing the podocytes, proximal tubule, Henle’s loop, and distal tubule [3].

In this in vivo study, we examined fetal lamb kidneys using immunohistochemistry to determine the location and timing of the expression of transcription factors WT1, HNF1β, Pax2, and Pax8, which have been reported to be involved in renal development.

## 2. Materials and Methods

After approval was obtained from the Animal Ethics Committee of the Wellington School of Medicine and Health Sciences, University of Otago, Wellington, New Zealand (approval numbers WAEC 8-03, W03/07, W02-11, W01-12, AEC4-14, AEC 1-16), pregnant ewes were transported from the farm 24 to 48 h before the operation. They were examined by ultrasound to confirm the pregnancy and to avoid unnecessary operations.

Our preoperative and anesthetic management was reported previously [4]. At 50, 60, 70, 80, 90, 100, and 110 days, and full term (145 days), the ewes were anesthetized, and the fetuses were delivered by cesarean section. The lambs were then sacrificed using pentobarbital injected into the umbilical vein, as we previously described [5]. The lambs’ kidneys were then removed and fixed in 10% formalin. The kidneys were divided longitudinally, and samples were taken from the cut surface of the kidney, processed for light microscopy in paraffin blocks, and cut into 3 μm slices for light microscopy. Sections were deparaffinized in xylene and rehydrated in a graded series of alcohol followed by H_2_O_2_. The sections were then either stained with hematoxylin and eosin (HE) or immunohistochemistry. For immunohistochemistry, we used WT1 mouse monoclonal antibody, NCL-WT1-562 (Leica, Wetzlar, Germany), 1:20 dilution, 60 min; anti-HNF1β rabbit polyclonal antibody, HPA002083 (Sigma-Aldrich, St. Louis, MO, USA), 1:1500 dilution, 60 min; anti-Pax8 mouse monoclonal antibody, ab53490 (Abcam, Cambridge, UK), 1:10 dilution, 60 min; and anti-Pax2 rabbit monoclonal antibody, ab70389 (Abcam, Cambridge, UK), 1:500 dilution, 60 min.

## 3. Results

### 3.1. Number of Samples

Fetal lamb kidneys at 50 (*n* = 4), 60 (*n* = 5), 70 (*n* = 2), 80 (*n* = 2), 90 (*n* = 4), 100 (*n* = 5), 110 (*n* = 4), and 145 (full term, *n* = 4) days of gestation and adult ewe kidneys (*n* = 3) were included in this study. Because the fetal kidneys at 50, 60, and 70 days of gestation were very small, the largest cut surfaces of both kidneys were used for the evaluation, while slices from the left kidney were used for the evaluation for fetuses at 80, 90, 100, and 110 days and full term.

### 3.2. Morphological Changes in the Kidney in Fetal Lambs

**Figure 1:**
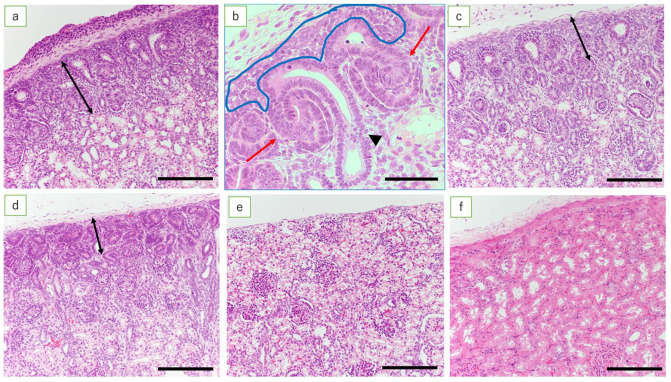
Nephrogenic zone (HE). (**a**) Fetal kidney at 50 days’ gestation (HE, scale bar: 200 μm); (**b**) fetal kidney at 50 days’ gestation (HE, scale bar: 100 μm). Metanephric mesenchymal cells (inside the blue line), ureteric bud (arrowhead), and S-shaped bodies (red arrows); (**c**) fetal kidney at 70 days’ gestation (scale bar: 200 μm); (**d**) fetal kidney at 110 days’ gestation (scale bar: 200 μm); (**e**) fetal kidney at term (scale bar: 200 μm); and (**f**) the ewe’s kidney (scale bar: 200 μm). Fetal kidney at 50 days’ gestation has NZ in the renal cortex and the ureteric bud, metanephric mesenchymal cells, and C- and S-shaped bodies. As pregnancy progresses, there is progressive thinning of the NZ, resulting in the loss of NZ at full term. The adult ewe kidney after the development of renal tubules is complete shows well-established tubular epithelial cells and increased inter-glomerular distance (double arrows in (**a**–**d**) indicate the extent of the NZ).

Beneath the renal capsule of the fetal lamb kidneys at 50 days, the nephrogenic zone (NZ), ureteric bud, metanephric mesenchymal cells, and C- and S-shaped bodies were observed. In addition, mature glomeruli and renal tubules were found in the deep cortex and near the medulla (data not shown). The NZ in kidneys was found in fetuses at 50, 60, 70, 80, 90, 100, and 110 days of gestation; the NZ thinned progressively as the gestational age increased. No NZ was found in the kidneys of full-term lambs (Figure 1).

### 3.3. Immunohistochemistry

**Figure 2:**
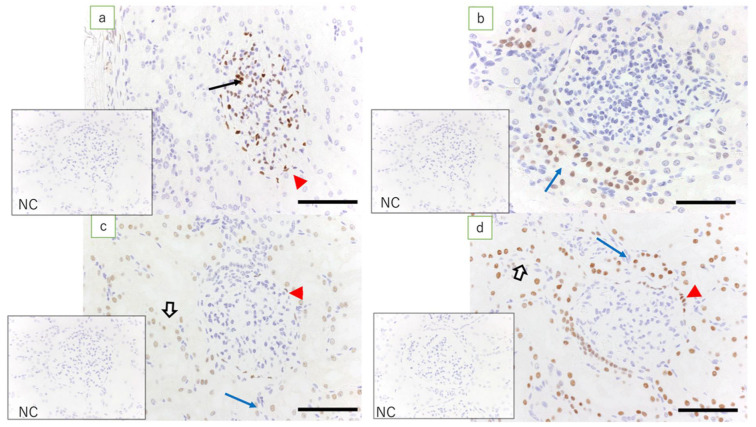
Ewe’s kidney (immunostaining, scale bar: 100 μm). (**a**) Expression of WT1 is found in glomerular epithelial cells (arrow) and epithelial cells of Bowman’s capsule (red arrowhead); (**b**) low expression of Pax2 is found only in the collecting duct epithelial cells (blue arrow); (**c**) expression of Pax8 is found in epithelial cells of Bowman’s capsule (red arrowhead), tubular epithelial cells (white arrow), and epithelial cells in the collecting duct (blue arrow); and (**d**) expression of HNF1β, which is equivalent to that in the kidney in term lambs, is found in epithelial cells of Bowman’s capsule (red arrowhead), tubular epithelial cells (white arrow), and epithelial cells in the collecting duct (blue arrow); NC: negative control.

In the adult ewe kidney (control), the expression of WT1 was noted only in the glomerular epithelial cells and epithelial cells of the Bowman’s capsule, whereas low expression of Pax2 was found only in the epithelial cells in the collecting duct. Pax8 was expressed in tubular epithelial cells, in the epithelial cells of Bowman’s capsule, and in the epithelial cells of the collecting duct. Expression of HNF1β was found in the proximal and distal tubular epithelial cells, in the epithelial cells of Bowman’s capsule, and in the epithelial cells in the collecting duct (Figure 2).

**Figure 3:**
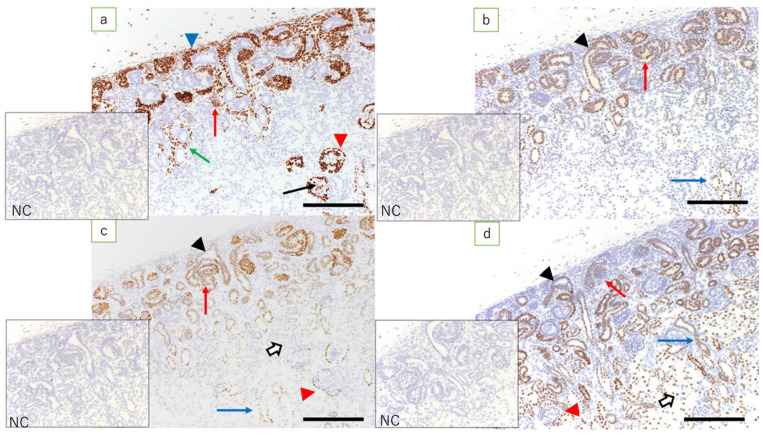
Fetal kidney at 90 days’ gestation (immunostaining, scale bar: 200 μm). (**a**) Expression of WT1 is found in metanephric mesenchymal cells, C- and S-shaped bodies (red arrow), glomerular epithelial cells (arrow), epithelial cells of Bowman’s capsule (red arrowhead), medullary rays (green arrow), and interstitial cells in the medulla (blue arrowhead); (**b**) expression of Pax2 is found in the ureteric bud (arrowhead), C- and S-shaped bodies (red arrow), and epithelial cells in the collecting duct (blue arrow); (**c**) expression of Pax8 is found in the ureteric bud (arrowhead), C- and S-shaped bodies (red arrow), tubular epithelial cells (white arrow), epithelial cells of Bowman’s capsule (red arrowhead), and epithelial cells in the collecting duct (blue arrow); (**d**) expression of HNF1β is found in the ureteric bud (arrowhead), C- and S-shaped bodies (red arrow), tubular epithelial cells (white arrow), some of the epithelial cells of Bowman’s capsule (red arrowhead), and epithelial cells in the collecting duct (blue arrow); NC: negative control.

**Figure 4:**
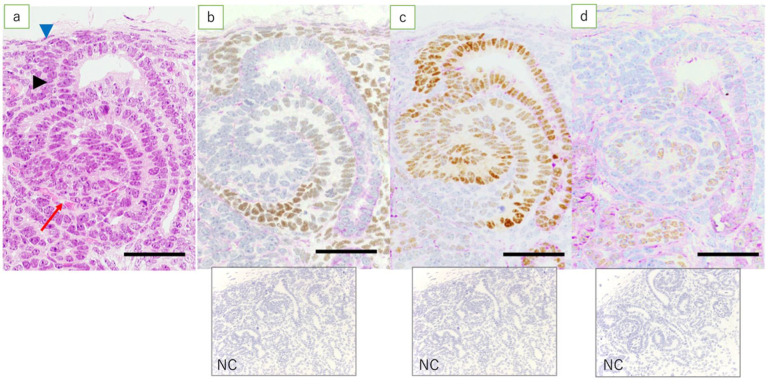
NZ in the fetal kidney at 90 days’ gestation. (HE, immunostaining, scale bar: 40 μm). (**a**) HE; metanephric mesenchymal cells (blue arrowhead), ureteric bud (arrowhead), S-shaped body (red arrow); (**b**) expression of WT1 is found in metanephric mesenchymal cells, renal vesicle, and S-shaped body, more proximal to the medulla; (**c**) expression of Pax2 is mostly found in the ureteric bud, renal vesicle, and S-shaped body; and (**d**) expression of HNF1β is found in the middle region, more proximal to the cortex in the S-shaped body than cells expressing WT1; NC: negative control.

**Figure 5:**
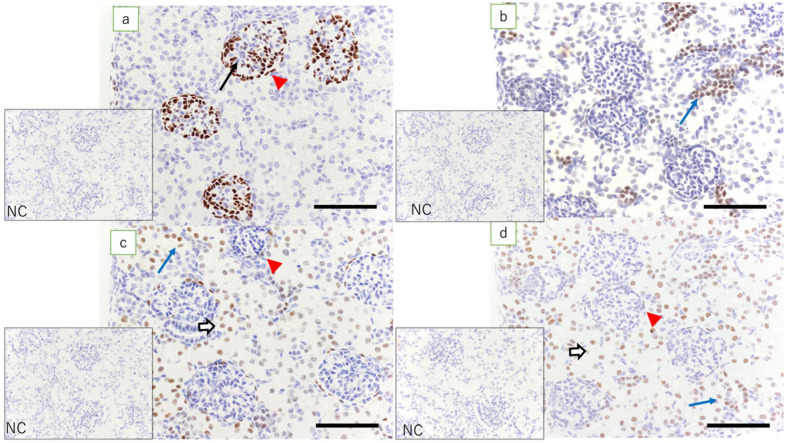
Fetal kidney at term (immunostaining, scale bar: 100 μm). The NZ has been completely lost. (**a**) Expression of WT1 is found in glomerular epithelial cells (arrow) and epithelial cells of Bowman’s capsule (red arrowhead); (**b**) expression of Pax2 is found in the epithelial cells in the collecting duct; (**c**) expression of Pax8 is found in tubular epithelial cells (white arrow), epithelial cells of the Bowman’s capsule (red arrowhead), and epithelial cells in the collecting duct (blue arrow); and (**d**) expression of HNF1β is found in epithelial cells of Bowman’s capsule (red arrowhead), tubular epithelial cells (white arrow), and epithelial cells in the collecting duct (blue arrow); NC: negative control.

In the fetal kidneys at ≤110 days of gestation where an NZ was observed, the expression of WT1 was found in metanephric mesenchymal cells, C- and S-shaped bodies, glomerular epithelial cells, epithelial cells of Bowman’s capsule, the medullary rays, and in interstitial cells in the medulla (Figure 3a). Among these, the expression of WT1 in the renal vesicle and C- and S-shaped bodies was noted mainly in cells closer to the medulla (Figure 4b). In the kidneys of full-term fetuses with no NZ, expression of WT1 was found in glomerular epithelial cells, the epithelial cells of Bowman’s capsule, and the interstitial cells in the medullary rays (Figure 5a), in marked contrast with the findings in adult kidneys.

Fetal kidneys at ≤110 days of gestation revealed the expression of Pax2 in the ureteric bud, C- and S-shaped bodies, and epithelial cells in the collecting duct (Figure 3b). In lambs at 90 days’ gestation, expression of Pax2 was found in the middle and peripheral regions of the renal vesicle and C-shaped bodies, and in the S-shaped bodies proximal to the medulla (Figure 4c). In the kidneys of full-term fetuses, only epithelial cells in the collecting duct were positive, similar to the findings in the adult kidney (Figure 5b).

Fetal kidneys at ≤110 days of gestation revealed the expression of Pax8 in the ureteric bud, C- and S-shaped bodies, tubular epithelial cells, epithelial cells of Bowman’s capsule, and epithelial cells in the collecting duct (Figure 3c). The expression of Pax8 in renal vesicles and C- and S-shaped bodies did not show obvious polarity. In the kidneys of term fetuses, the expression of Pax8 was found in epithelial cells of Bowman’s capsule, tubular epithelial cells, and epithelial cells in the collecting duct (Figure 5c), similar to the findings in the adult kidneys.

In fetal kidneys at ≤110 days of gestation, the expression of HNF1β was found in C- and S-shaped bodies, tubular epithelial cells, some of the epithelial cells of Bowman’s capsule, and epithelial cells in the collecting duct (Figure 3d). In the renal vesicles and C- and S-shaped bodies, high expression of HNF1β was mainly noted, slightly more proximal to the renal capsule than cells expressing WT1 (Figure 4d). In the kidneys of full-term fetuses, the expression of HNF1β was found in the epithelial cells of the Bowman’s capsule, tubular epithelial cells, and epithelial cells in the collecting duct (Figure 5d), again similar to the findings in adult kidneys.

## 4. Discussion

WT1 was originally reported to be a causative gene in the genesis of nephroblastoma. However, it is mainly involved in the normal development of the kidney, spleen, and gonads. WT1 mutations may lead to nephroblastoma, glomerular sclerosis, gonadal dysgenesis, congenital diaphragmatic hernia, and cardiac disease. Mice with the loss of WT1 do not have kidneys or gonads, exhibit congenital diaphragmatic hernia, and die due to cardiac problems. In renal development, WT1 plays an essential role in MET (the basis of nephron differentiation), which initiates renal development [6]. HNF1β is a transcription factor that modulates the development and function of renal, hepatic, pancreatic, and urogenital epithelial cells. HNF1β is required for the branching of the ureteric bud, onset of kidney development, and nephron differentiation [7]. It was reported that the Pax gene family, consisting of nine genes, is involved in the development of the central nervous system, spine, eyes, and kidneys. It was found that Pax2 and Pax8 mainly contribute to the development of pronephros and mesonephros and that Pax2 and Pax8 double-null mice fail to generate the nephric duct and have renal dysplasia [8].

Recent in vitro studies of renal development have produced substantial advances, enabling the efficient creation of kidney-like tissue (kidney organoids) from hESCs and hiPSCs. Morizane et al. [3] found that the development of the nephron starts with the induction of T + TBX6 + (primitive streak) and WT1 + HOXD11 + Pax2 − LHX1 − (the posterior intermediate mesoderm). Although Pax2 and LHX1 are negative in the posterior intermediate mesoderm, they induce NPCs (SIX2 + SALL1 + WT1 + Pax2 +). The NPCs result in pretubular aggregates and Pax8, LHX1, and LAM-positive renal vesicles with the expression of HNF1β and BRN1, leading to the differentiation of epithelial cells [3]. The polarity in renal vesicles and C- and S-shaped bodies has started to be elucidated. In their lower pole, WT1 promotes the proximal differentiation (especially podocytes) by antagonizing Pax2, leading to the release of signals that attract endothelial cells. HNF1β affects the upper pole of the C-shaped bodies. In the S-shaped bodies, the expression of HNF1β found in the lower pole and middle regions and modulates the expression of notch ligands and Irx1/2, promoting proximal, intermediate, and medial processes [9,10]. In a study of the collecting duct system, Taguchi et al. [11] generated induced Wolffian duct progenitor cells with the expression of Pax2, LHX1, Emx1, Sim1, and Gata3 from pluripotent stem cells. They observed the expression of HNF1β, Wnt9b, and Calb1, which are markers of a mature ureteric bud. In the anterior intermediate mesoderm, the expression of Osr1, Pax2, Lhx1, and Pax8 was noted, leading to the development of the ureteric bud from the Wolffian duct progenitor cells. In the Wolffian duct progenitor cells, due to the loss of expression of markers of the metanephros, such as WT1, the expression of Pax2 mainly affects renal development [11]. HNF1β was found to be involved in the growth and differentiation of the ureteric bud [12].

Previous in vivo studies have also shown that WT1, Pax2, Pax8, and HNF1β play important roles in the formation of the posterior kidney, but do not provide a detailed distribution of cells [13,14,15]. Using immunohistochemistry, we examined the expression of these transcription factors, which play important roles in renal development in lamb fetuses. In kidneys at 60 days of gestation, high expression of WT1 was noted in the mesenchyme around the ureteric bud. The expression of WT1 was also found in glomerular epithelial cells and the epithelial cells of Bowman’s capsule. In renal vesicles and C- and S-shaped bodies, high expression of WT1 was observed in the lower pole. These results suggest that the expression of WT1 around the ureteric bud is involved in the development of the metanephric mesenchymal cells into the epithelial cells of Bowman’s capsule. The loss of WT1 in the epithelial cells of Bowman’s capsule and the surrounding tubular epithelial cells suggests the involvement of WT1 mainly in the development of podocytes in the glomerulus and the parietal epithelium of Bowman’s capsule. During early gestation, the expression of Pax2 and Pax8 was observed in the ureteric bud and S-shaped bodies but not in glomerular epithelial cells. In fetal kidneys of all gestational durations, the expression of Pax2 was not observed in Bowman’s capsule epithelial cells or tubular epithelial cells, but the expression of Pax8 was detected. The expression of Pax8 was maintained in an adult ewe’s kidneys, suggesting that the expression of Pax8 is involved in the function of epithelial cells of the Bowman’s capsule and the tubular epithelial cells. In early gestational lamb kidneys, high expression of HNF1β was found in the ureteric bud and S-shaped bodies. At later gestational time points, high expression of HNF1β was noted in the renal tubules and collecting ducts. In renal vesicles and C- and S-shaped bodies, high expression of HNF1β was noted in cells in the upper pole and middle regions, slightly more proximal to the renal capsule than cells expressing WT1. Unlike WT1, this suggests the involvement of HNF1β in the development of the (peripheral) epithelium of the nephron or tubular epithelial cells. In addition, high expression of HNF1β in the collecting duct suggests that HNF1β is involved in the development of the nephron and the collecting duct system.

The above results show that the development of the nephron from metanephric mesenchymal cells to tubular epithelial cells starts with the activation of WT1, leading to its development into glomerular epithelial cells and epithelial cells of the Bowman’s capsule. In addition, the activation of HNF1β, Pax2, and Pax8 plays an important role in the differentiation of peripheral tubular epithelial cells. The results of the current study are consistent with previous in vitro studies using WT1 as a marker for the differentiation from undifferentiated stem cells into immature nephronal cells in which HNF1β, Pax2, and Pax8 expressed in sequence are used as markers of differentiation into podocytes, epithelial cells of Bowman’s capsule, and tubular epithelial cells [10]. Furthermore, our findings suggest that Pax2 and Pax8 may be involved in nephron development and the development of the ureteric bud into the collecting duct system.

The role of the co-expression of Pax2 and Pax8 in the development of the collecting duct system has not yet been elucidated. In early-gestation fetuses, it was found that Pax2 and Pax8 are expressed independently and that Pax2 knock-out mice never develop into the metanephros, whereas Pax8 knock-out mice show normal kidney development [15]. The postpartum expression of Pax2 persists only in the collecting duct, whereas the expression of Pax8 during early gestation and postpartum is found throughout the entire epithelium from Bowman’s capsule to the collecting duct. This suggests that Pax8 modulates selective transporters in the medulla to maintain the salt–water balance even in the postpartum period [16]. Future genetic research should elucidate the morphological features found in the current study of renal development.

In this study, the location of the expression of transcription factors in vivo that have been reported to be involved in renal development in in vitro studies was clarified. However, this study provides a morphological point of view of cells, and genetic studies are required to determine its specific role, which is the limitation of this study. Each of these cells, consisting of the glomerulus, Bowman’s capsule, renal tubules, and collecting ducts, in mature kidney tissue may have a specific function, and the abovementioned transcription factors may be involved in the expression of these genes that are responsible for functional expression. Future studies on the relationship between transcription factors and the activation of these genes might help to elucidate the process of renal development.

In the studies of this area, models of the early embryonic period using cultured cells and small animals such as mice and rats have been examined [8,12,17]. However, in this study, the sheep model, which is a large animal, was used, and we compared and examined it with term models and ewes. It is necessary to examine a knock-out model for the study of gene expression, but this could not be done here because the method has not yet been established for sheep.

## 5. Conclusions

Immunohistochemistry for WT1, HNF1β, Pax2, and Pax8 may help with evaluating the transcription factors in renal development. Our findings suggest the possibility that WT1 is mainly involved in development of the nephron, whereas HNF1β, Pax2, and Pax8 are involved in the maturation of the nephron and development of the Wolffian duct that branches to form the peripheral collecting ducts.

## Data Availability

Not applicable.
